# Causal association study of the dynamic development of the metabolic syndrome based on longitudinal data

**DOI:** 10.1038/s41598-024-55693-3

**Published:** 2024-03-05

**Authors:** Jaina Razbek, Liangliang Bao, Yan Zhang, Mayisha Daken, Mingqin Cao

**Affiliations:** 1 Department of Epidemiology and Health Statistics, College of Public Health, Xinjiang Medical University, Urumqi, China; 2 Department of Postgraduate Management Section, The Fourth Affiliated Hospital of Xinjiang Medical University, Urumqi, China; 3 Department of Epidemic Prevention, Karamay Centre for Disease Control and Prevention, Karamay, China

**Keywords:** Endocrinology, Risk factors

## Abstract

The dynamic progression of metabolic syndrome (MetS) includes developmental deterioration and reverse recovery; however, the key factors in this bidirectional progression have not been identified. Our study aimed to use the data obtained from the China Health and Retirement Longitudinal Study (CHARLS) and construct a Bayesian network to explore the causal relationship between influential factor and the development and recovery of MetS. Followed up at 4 years, forward progression of MetS occurred in 1543 and reverse recovery of MetS occurred in 1319 of 5581 subjects. Bayesian Networks showed that hyperuricemia and body mass index (BMI) levels directly influenced progression of MetS, and gender, exercise and age play an indirect role through hyperuricemia and BMI levels; high hemoglobin A1c (HbA1c) and BMI levels directly influenced recovery of MetS, and gender and exercise play an indirect role through BMI levels. Bayesian Network inference found that the rate of progression of MetS in subjects with hyperuricemia increases from 36 to 60%, the rate of progression of MetS in subjects with overweight or obese increases from 36 to 41% and the rate of reverse recovery rate of MetS in subjects with high HbA1c decreased from 33 to 20%. Therefore, attention to individuals at high risk of hyperuricemia, high HbA1c levels, and overweight/obesity should be enhanced, with early detection and following healthy behavioral interventions to prevent, control and delay the progression of MetS and its components.

## Introduction

The occurrence of Metabolic syndrome (MetS) is not be achieved overnight. Its essence is the combined synergistic effect of abnormally clustered cardiovascular risk factors and individual, environmental and other factors have a combined synergistic effect^1,2^, which not only seriously affects individual organs, but also is related to cardiovascular disease, type 2 diabetes and even death^3,4^. Under the joint action of complex multi-factors, MetS does not follow a continuous progression pattern^5^, and there is not only a development deterioration but also a reverse recovery^6^. Identifying key factors in the development and recovery of MetS will facilitate the development of interventions and other targeted measures to control or even reverse the development of MetS, which has public health implications for the prevention of MetS and related chronic diseases. Among these, the focus needs to be on the fact that researchers attempting to screen for intervenable factors for preventive interventions and treatments should obtain evidence based on causal inference, and causal evidence is the evidence-based basis for making decisions about disease interventions^7^.

However, most studies have focused on exploring the occurrence and development process of MetS and its associated influencing factors^8–12^. Currently, there are divergent opinions and no consensus in the medical field regarding its key factors, etiology and pathogenesis^13^. Therefore, there is still a need to carry out studies on the key factors in the development and recovery of MetS adapted to the clinical reality by making rational use of the methodology, so as to provide information on the evidence that can be intervened. Bayesian networks^14^ can describe the causal relationships between variables and solve real-world uncertainty aspects through qualitative and quantitative, with intuitive forms, clear semantics, and comprehensibility, and their effectiveness has been proven in many fields. The method assigns conditional probabilities on the basis of directed acyclic diagrams, revealing a reticulated association between each influencing factor and the critical event, allowing not only to identify the direct influencing factors with causal effects on the critical event, but also to understand the complex joint and synergistic interactions among the factors^15^.

Our Subject Group used longitudinal data from the China Health and Retirement Longitudinal Study (CHARLS), previous study that analyzed the influencing factors associated with the positive progression and reversal of MetS, and concluded that the gender, age, exercise, smoke, drink, hyperuricemia, high HbA1c, functional loss, and BMI level acted as the key factors influencing their history of disease progression^10^. Therefore, on this basis, the above influencing factors associated with positive MetS progression and reversal were used as candidate variables in the causal inference model. The causal relationship between the influencing factors and disease progression/recovery was visualized through Bayesian network modelling for further causal and evidential inference, thus providing evidence to support the development of preventive intervention strategies to halt or even reverse the progression of MetS.

## Results

### Dynamic progression of the MetS

This study included 5581 subjects, of whom 2100 were males and 3481 were females. At baseline, there were 2479 patients with MetS, 1819 males and 660 females, and 3102 without MetS, 1662 males and 1440 females. All them were followed up at 4 years, there were 2572 patients with MetS, 1803 males and 769 females, 863 new cases of MetS, and the cumulative incidence rate of MetS was 27.82%. Disease status remained unchanged in 2719 cases (48.72%) in the survey population. forward progression of MetS occurred in 1543 people (53.91% of the number of changes) and reverse recovery of MetS occurred in 1319 cases (46.09% of the number of changes).

### Bayesian network learning of the forward progression of MetS and key factors

**Figure 1:**
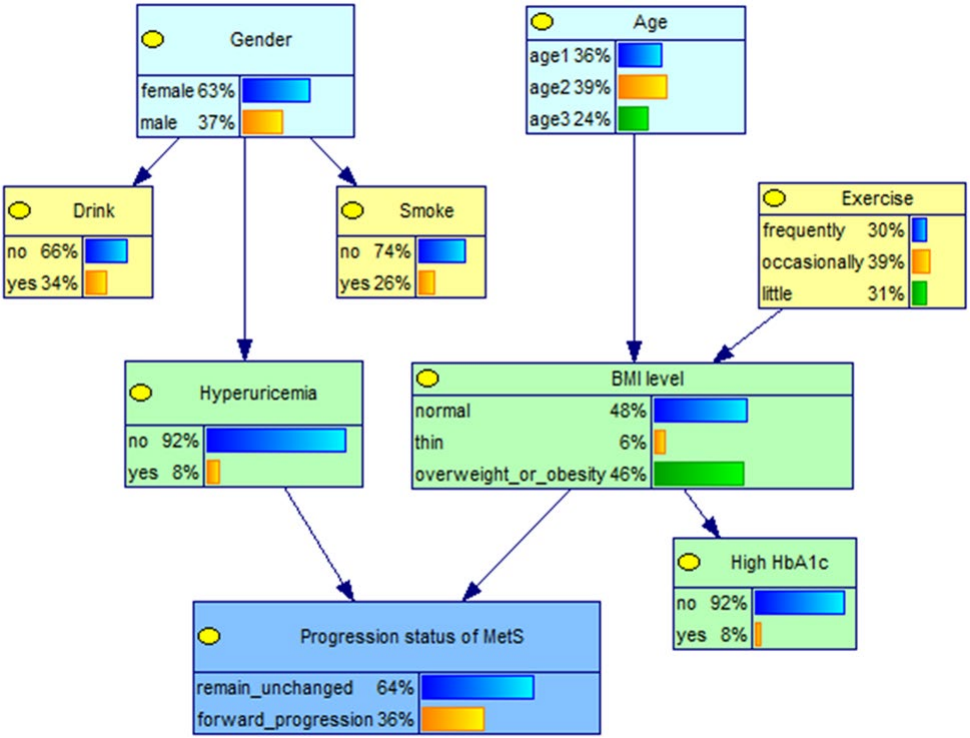
Edge probability distribution of each nodes in Bayesian network model of progression status of MetS.

The DAG structure retains 9 nodes and 8 edges. The joint probability values and the network structure are shown in Fig. 1. MetS progression status has 2 parent nodes, hyperuricemia and body mass index (BMI) levels, which are direct influences on the forward progression of the MetS. Meanwhile, gender, age and exercise status are indirectly influencing the forward development of MetS by affecting other factors, of which gender directly influences hyperuricemia and thus plays an indirect role; age and exercise status indirectly influence the occurrence of forward progression of the MetS through BMI level.

The conditional probabilities of each node are shown in Table 1. Depending on the combination of six different value levels of the parent node (hyperuricemia, BMI level), the child nodes (MetS progression status) will correspond to six different conditional probability values. For example, the probability of forward progression of MetS in an individual with hyperuricemia and a BMI level at overweight or obese is 71.99%.

**Table 1:** The conditional probability distribution of progression status of MetS.

Hyperuricemia	BMI level	Remain unchanged (%)	Forward progression (%)
No	Normal	70.61	29.39
	Thin	65.73	34.27
	Overweight or obesity	62.00	38.00
Yes	Normal	45.00	55.00
	Thin	94.68	5.32
	Overweight or obesity	28.01	71.99

### Causal and evidential reasoning for the forward progression of MetS and key factors

**Figure 2:**
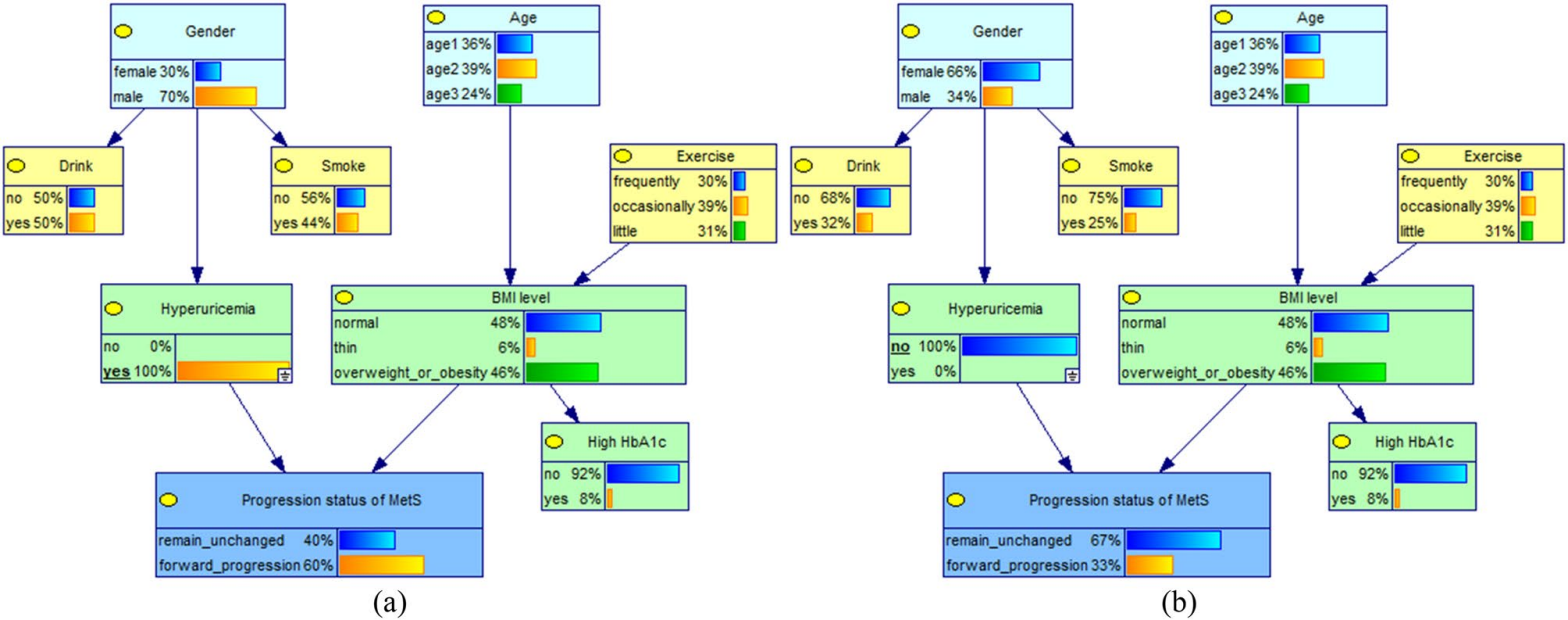
Conditional probability changes of the progression status of MetS in different hyperuricemia conditions (**a**) subjects with Hyperuricemia; (**b**) subjects without Hyperuricemia.

**Figure 3:**
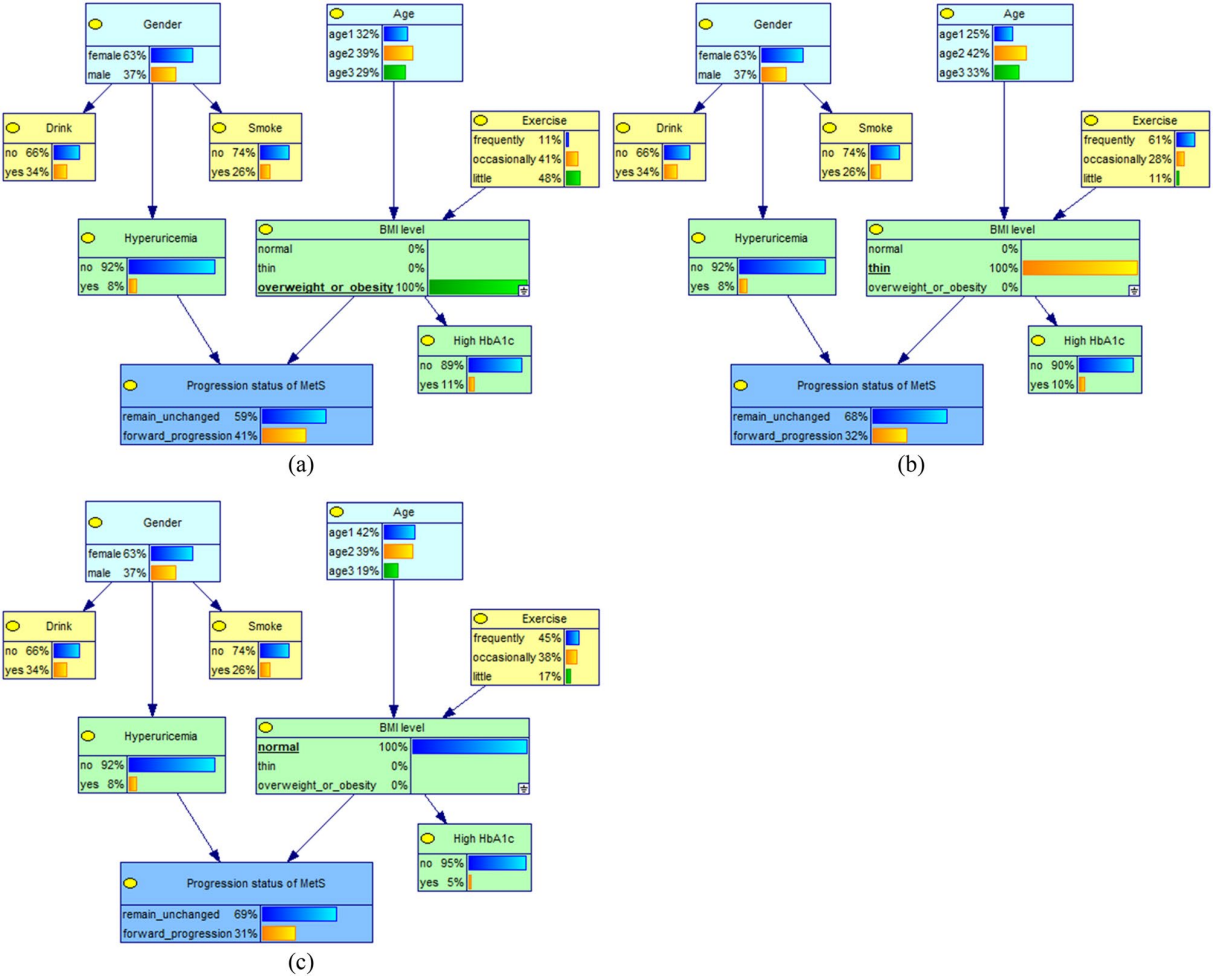
Conditional probability changes of the progression status of MetS in different BMI levels. (**a**) subjects with overweight or obesity; (**b**) subjects with thin weight; (**c**) subjects with normal weight.

When the independent variable is known, the corresponding parent node's value level in the network can be adjusted accordingly, and then the posterior probability of the child node (progression status of MetS) can be observed to infer the relationship between the variables, as shown in Figs. 2 and 3. If the value level of "hyperuricemia" is known to be "yes", and the status is set to suffer from hyperuricemia (100%), after updating the network, the rate of forward progression of MetS increases from 36 to 60%, which is an increase of 24%; when the status was set to not suffering from hyperuricemia (100%), the forward progression rate decreased from 36 to 33%.

When the probability of BMI level "overweight or obese" was 100%, the rate of forward progression of MetS increased from 36 to 41%; when the probability of "thin" was 100%, the forward progression rate decreased from 36 to 32%; when the probability of "normal" was 100%, the forward progression rate decreased from 36% to 31%. In addition, When the probability of "male" of gender, ">65 years" of Age and "little" of exercise was 100%, the rate of forward progression of MetS all increased from 36% to 38%; This suggests that the rate of forward progression of MetS was higher in subjects with hyperuricemia, overweight or obesity, male, >65 years and little exercise.By using the evidential inference function of the Bayesian network, the probability changes of the influencing factors were compared when the progression status of MetS was remain unchanged or forward progression, as shown in Table 2. When the progression status of MetS was forward progression, the probability of its direct influence factors obesity or overweight increased from 46.37% to 53.05% and hyperuricemia increased from 8.41 to 14.24%, thus confirming that overweight or obesity and hyperuricemia are the direct causes influencing the forward progression of MetS. In addition, the probability of little exercise increased slightly from 31.33 to 33.21%, male from 37.06 to 39.06% and ≥65 year from 24.41 to 25.01%. It is suggested that these factors can influence the forward progression of MetS through BMI level and hyperuricemia, respectively.

**Table 2:** Changes in the probability distribution of factors under different progression status of MetS(%).

Variables		Probability distribution (%)	Forward progression (%)	Remain unchanged (%)
Gender	Female	62.94	60.94↓	64.11↑
	Male	37.06	39.06↑	35.89↓
Age	40 ~	36.32	35.78↓	36.58↑
	55 ~	39.27	39.21↓	39.32↑
	≥ 65	24.41	25.01↑	24.10↓
Exercise	Frequently	30.15	27.64↓	31.32↑
	Occasionally	38.52	39.15↑	38.86↑
	Little	31.33	33.21↑	29.82↓
BMI level	Normal	47.91	41.92↓	51.32↑
	Thin	5.72	5.03↓	6.11↑
	Overweight or obesity	46.37	53.05↑	42.57↓
Hyperuricemia	Yes	8.41	14.24↑	5.28↓
	No	91.59	85.76↓	94.72↑

### Bayesian network learning of the reverse recovery of MetS and key factors

**Figure 4:**
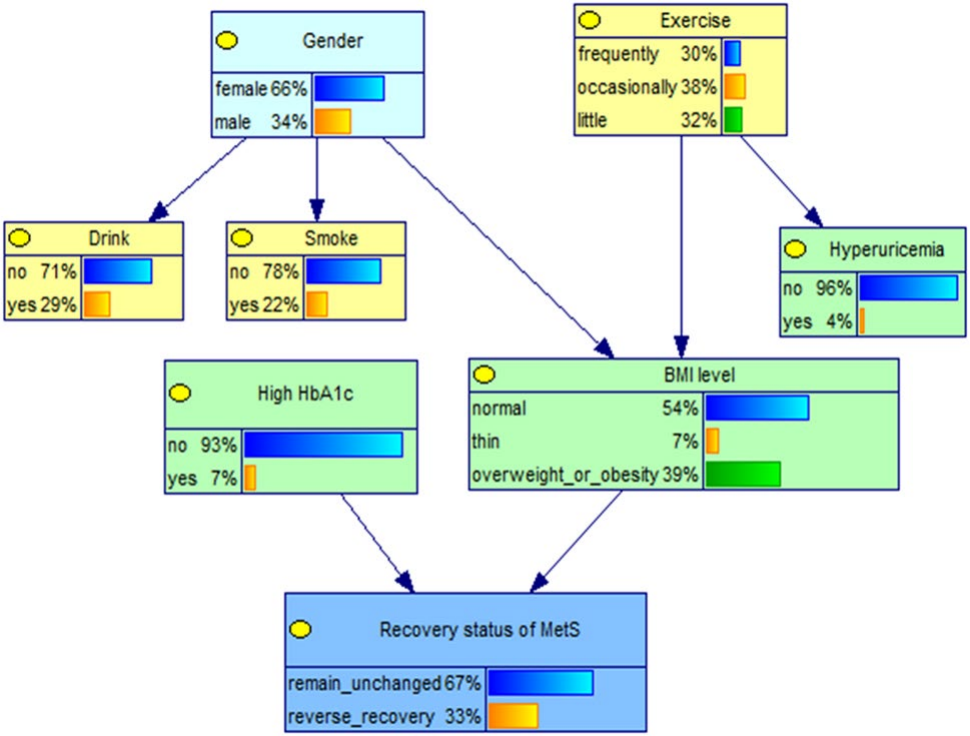
Edge probability distribution of each nodes in Bayesian network model of recovery status of MetS.

The DAG structure of the reverse recovery of MetS and key factors retained 8 nodes and 7 edges (shown in Fig. 4), the network structure shows that the reverse recovery of MetS condition has 2 parent nodes, which are high hemoglobin A1c (HbA1c) and BMI level, that is, they are the direct influencing factors. Meanwhile, gender and exercise status are indirectly influencing the reverse recovery of MetS by affecting the BMI level.

The conditional probability table of each network node was obtained by parameter learning, which is shown in Table 3. Depending on the six different combinations of value levels of the parent node (high HbA1c, BMI level), the child nodes (recovery status of MetS) will correspond to six different conditional probability values. The probability of reverse recovery of MetS was 45.65% in those who did not suffer from high HbA1c and were thin.

**Table 3:** The conditional probability distribution of recovery status of MetS.

High HbA1c	BMI level	Remain unchanged (%)	Reverse recovery (%)
No	Normal	65.67	34.33
	Thin	54.35	45.65
	Overweight or obesity	69.08	30.92
Yes	Normal	75.33	24.97
	Thin	73.35	26.65
	Overweight or obesity	87.63	12.37

### Causal and evidential reasoning for the reverse recovery of MetS and key factors

**Figure 5:**
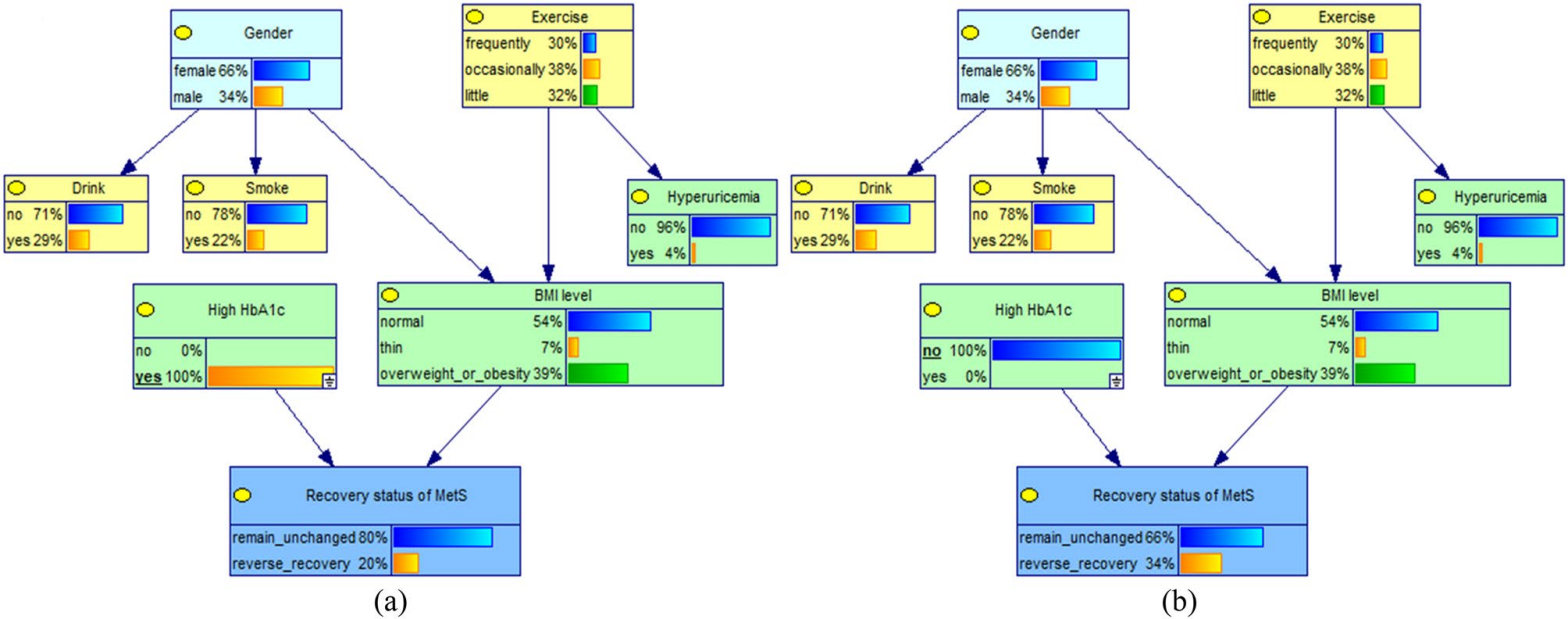
Conditional probability changes of the recovery status of MetS in different HbA1c conditions. (**a**) subjects with high HbA1c; (**b**) subjects without high HbA1c.

When the variable "high HbA1c" was instantiated as "no", the rate of reverse recovery of MetS was observed to increase slightly from 33 to 34%; When the "high HbA1c" variable was instantiated as "yes", the reverse recovery rate decreased from 33 to 20%, as shown in Fig. 5.

**Figure 6:**
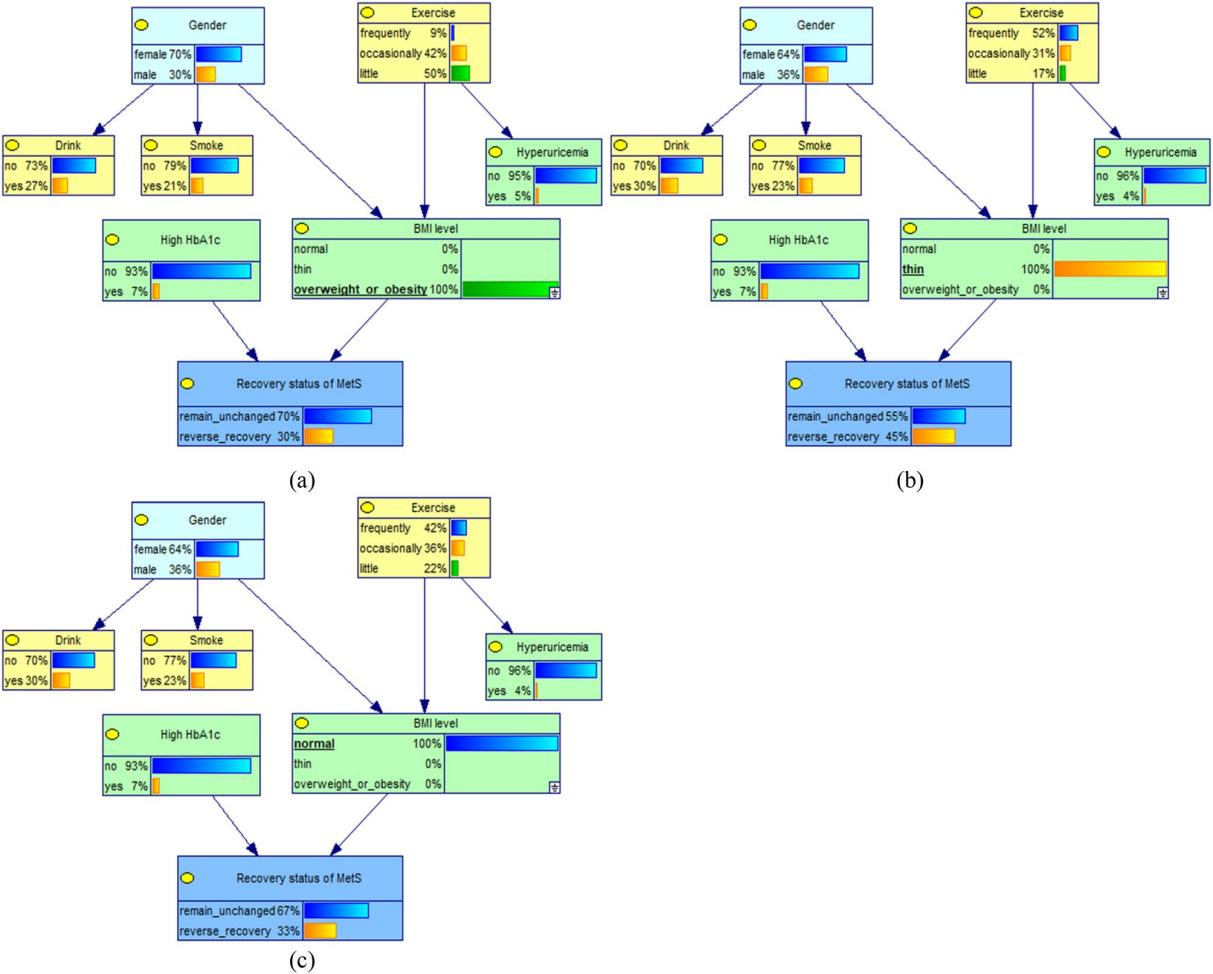
Conditional probability changes of the recovery status of MetS in different BMI levels. (**a**) subjects with overweight or obesity; (**b**) subjects with thin weight; (**c**) subjects with normal weight.

When the probability of "thin" of BMI level was 100%, the rate of reverse recovery of MetS increased from 33 to 45%; when the probability of "normal" of BMI level was 100%, the reverse recovery rate remained unchanged; however, when the probability of "overweight or obese" was 100%, the reverse recovery rate decreased from 33 to 30%. This suggests that high HbA1c and BMI levels are important factors affecting the reverse recovery of MetS, as shown in Fig. 6. In addition, When the variable "gender" was instantiated as "femal or male", the reverse recovery rate remained unchanged; When the variable " exercise " was instantiated as " little", the rate of reverse recovery of MetS was observed to decrease slightly from 33 to 32%. This suggests that high HbA1c and BMI levels are important factors affecting the reverse recovery of MetS,

When the recovery status of MetS was reverse recovery, the probability of BMI level being normal and thin increased from 53.84 and 7.01% to 55.05 and 9.62%, respectively, and the probability of high HbA1c decreased from 7.28 to 4.42%, thus confirming that BMI level and high HbA1c were the direct causes influencing the reverse recovery of MetS. In addition, the probability of frequently exercise increased slightly from 29.57 to 31.08% and in men from 33.89 to 34.08%. It is suggested that exercise status and gender factors indirectly affect the reverse recovery of MetS through BMI level, as shown in Table 4.

**Table 4:** Changes in the probability distribution of factors under different recovery status of MetS(%).

Variables		Probability distribution (%)	Reverse recovery (%)	Remain unchanged (%)
Gender	Female	66.11	65.92↓	66.22↑
	Male	33.89	34.08↑	33.78↓
Exercise	Frequently	29.57	31.08↑	28.85↓
	Occasionally	38.16	37.82↑	38.32↑
	Little	32.27	31.10↓	32.83↑
BMI level	Normal	53.84	55.05↑	53.38↓
	Thin	7.01	9.62↑	5.70↓
	Overweight or obesity	39.15	35.33↓	40.92↑
High HbA1c	Yes	7.28	4.42↓	8.64↑
	No	92.72	95.58↑	91.36↓

## Discussion

Using longitudinal data from the CHARLS survey, there were 863 new cases of MetS with a cumulative incidence of 27.82% after 4 years of follow-up. Approximately more than half (51.28%) of the study subjects had a shift in disease status of MetS, of which it is of interest that 46.09% had a reverse recovery of MetS and 53.91% had forward progression of MetS. The diagnosis of MetS is complex and is a clustering situation of five components, but the disease may be reversible^16^. In addition to focusing on new cases, we need to focus on the progression of the disease and its recovery. The proportion of reverse reversal is comparable to the proportion of forward progression, further suggesting that whatever the baseline status of the study subjects is, it may improve. Therefore, follow-up observation of MetS should be strengthened and it is crucial to study the key factors that can be intervened and controlled in disease progression and recovery.

Based on the multi-state Markov models of dynamic progression of MetS in previous studies^10^, we found that obesity or overweight, physical inactivity, smoking, alcohol consumption and hyperuricaemia increase the risk of forward progression of MetS, females, obesity/overweight, high HbA1c, history of alcohol consumption, and low physical activity are all risk factors that impede recovery from MetS. On this basis, this study will further confirm the causal relationship between these factors and disease progression and recovery using Bayesian network modelling. From a methodological point of view, causal inference based on the results of previous studies, effectively improve the stability and causal inference efficacy of the Bayesian network model, thus proposing evidence-based information for intervention strategies to prevent and treat diseases. It was confirmed by Bayesian network learning and network inference that hyperuricemia, overweight/obesity is the direct cause of promoting the progression of MetS and high HbA1c, overweight/obesity is the direct cause of delaying disease recovery. The reason for this is that the hyperuricemic state causes oxidative stress, inflammatory response^17^ thereby inducing insulin resistance^18^, which may be the key to promoting the development of MetS. The development of obesity is closely related to inflammation and adipokines, where the inflammatory response is involved in the pathogenesis of the MetS, and adipose tissue, as an endocrine organ, disrupts the balance of pro- and anti-inflammatory factors secreted by adipokines in the inflammatory state, interfering with insulin signaling pathways and leading to insulin resistance^19^; on the other hand, it may lie in the genes related to lipid metabolism, obesity and insulin resistance and MetS and single nucleotide polymorphisms^20^, thus contributing to the occurrence and progression of the MetS. In addition to blood glucose, studies suggest that HbA1c levels can be an option for MetS screening^21^, due to the ability of HbA1c levels to effectively identify individuals at risk for MetS in people with normal fasting glucose^22^. Thus, during MetS screening, attention to individuals with hyperuricemia, high HbA1c levels, and overweight/obesity should also be enhanced, and the above groups should be considered as high-risk groups to further explore their risk factor thresholds for early detection and preventive intervention and treatment, thus preventing healthy, metabolically disturbed individuals from developing MetS.

It has been found that the influencing factors are not in parallel and can influence the forward progression and reverse recovery of the MetS by interacting with each other. Among them, gender and age non-modifiable factors indirectly influence the progression of MetS through hyperuricemia and BMI levels, and the evidence reasoned that men and advanced age are at high risk of forward progression of MetS. However, the results in the causal association analysis between the influencing factors and the reverse recovery of MetS showed difficult recovery in women. Although studies have reported higher prevalence of MetS in women^23^ and prevalence of each component^24^ than in men, studies on the natural history of MetS suggest^6,8,9^ that the rate of MetS development is higher in men than in women and that the prevalence and aggregation of each component of MetS may be higher in men than in women^25^, and this difference may be explained by the aging process that metabolic changes and changes in hormone levels differ significantly between individuals of different sexes^26,27^. Subsequently, taking into account the difficulty of recovery in females and the rapid progression of the disease in males, it is necessary to strengthen regular screening and at the same time apply preventive treatment to control the development of MetS.

Studies have shown that individuals with adverse behavioral patterns, such as sedentary^28^, high-fat diet^29^, smoking and alcohol consumption^10^, have a high prevalence of MetS, and that most of these adverse lifestyle conditions occur simultaneously^30^. The Bayesian network model found that exercise status indirectly influences the progression and recovery of MetS by directly affecting BMI levels. This is similar to the fact that individuals with less exercise have a higher risk of developing obesity^31^ and MetS^32^, and abdominal obesity is one of the important diagnostic criteria for MetS. Especially for people with abnormal BMI, exercise should be intensified to reduce insulin resistance and inflammatory response to control the progression of MetS. Behavioral interventions have been shown to improve not only the occurrence of MetS and various metabolic disorder components^33,34^, but also to reduce the incidence of diabetes and cardiovascular disease^35^. Thus, the most effective strategy that should be followed to prevent and delay the progression of MetS and its components is to change personal behavior and maintain healthy lifestyle habits^36^.

Based on CHARLS, this study uses nationally representative longitudinal data and a Bayesian network to better explain the causal relationship between key factors and progression and recovery from MetS. Facilitate the precise detection of individuals at high risk of MetS, and can provide evidence-based information for the early prevention, control and intervention of MetS. In addition, this study still has limitations: first, the study was a single follow-up at an interval of 4 years, making the precision of the model results limited, and multiple follow-ups are still needed to obtain larger and longer-term prospective studies to make the study provide stronger evidence. Second, the Bayesian network model established to explore the causal effect between baseline influences and disease progression and recovery in MetS, although with causal inference capability, with regard to the variables of interest, there are some relatively low proportions (probabilities). In the future, it will still be necessary to build dynamic Bayesian networks with sufficient sample size of dynamic data for further research.

## Conclusion

This study used a Bayesian network model to reveal the direct, indirect factors influencing the progression and recovery of MetS in a combined graphical plus conditional probability visualization. The study supports a causal relationship between hyperuricemia, high HbA1c and BMI levels and dynamic progression of the MetS, while gender, exercise status and age have indirect effects through the above factors. Therefore, attention to individuals at high risk of hyperuricemia, high HbA1c levels, and overweight/obesity should be enhanced, with early detection and following healthy behavioral interventions to prevent, control and delay the progression of MetS and its components.

## Methods

### Data source

The data for this study were obtained from the CHARLS survey conducted by the National Development Institute of Peking University which is an ongoing nationally representative longitudinal survey in China^37^. The survey includes assessments of basic demographics, health status, physical measurement, etc., Blood samples from the study population were collected in 2011 and 2015 through a standardized process that is described in detail on the website (http://charls.pku.edu.cn/).

This study selected 2011 baseline and 2015 follow-up data of the CHARLS to explore possible causes in the longitudinal development and recovery process of MetS by downloading modules on biomarkers, blood, demographic background and health status. The ID merge resulted in 5581 valid cases through the strict exclusion process. The detailed inclusion and exclusion of study subjects have been reported in our previous studies^10^. The previous study was conducted to analyse the influencing factors on the dynamic development of MetS in 5581 study subjects, but the influencing factors were not necessarily causally related. Thus, the present study was conducted in the same population in order to further investigate whether there is a causal relationship between the relevant influencing factors and the MetS disease state.

### Definition of metabolic syndrome development and recovery

**Figure 7:**
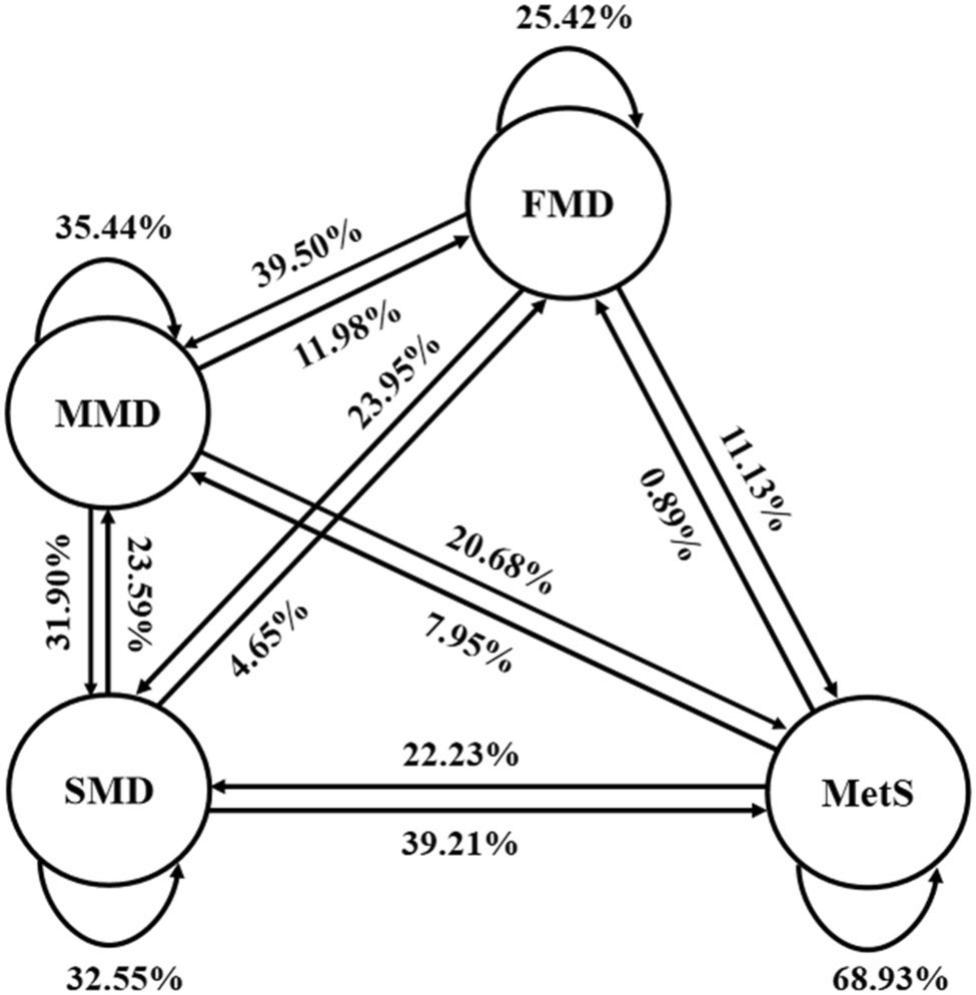
Structure chart of Metabolic syndrome dynamic progression.

Based on the definition of five metabolic component abnormalities the Joint Interim Statement^38^:①central obesity: waist circumference ≥85cm for men and ≥80cm for women ② hypertension: SBP/DBP ≥130/85 mmHg or those who have been diagnosed with and treated for hypertension; ③high fasting glucose: FBG ≥5.6 mmol/L or those who have been diagnosed with and treated for high glucose; ④ hypertriglyceride: TG ≥1.7 mmol/L; ⑤ low high density lipoprotein cholesterol: <1.03 mmol/L in men and <1.29 mmol/L in women, or those who have received treatment for this condition. Disease states were classified into four disease states: free of metabolic disorder(FMD, no abnormal component), mild metabolic disorder(MMD, one abnormal component), severe metabolic disorder(SMD, two abnormal components) and MetS (Three or more abnormal components)^10^. The dynamic transition is shown in Figure 7. Where disease progression is the forward progression of the MetS, including FMD →MMD, SMD and MetS; MMD →SMD and MetS; SMD →MetS. Disease improvement as a reverse recovery of the MetS, including MetS →SMD, MMD and FMD; SMD →MMD and FMD; MMD →FMD.

### Bayesian network

In this study, a Bayesian network model was constructed using whether the MetS undergoes forward progression or reverse reversal as the dependent variables, and the key variables derived from the previous study^10^ as network nodes (Table 5), as a way to explore the causal association between factors and dynamic outcomes.

**Table 5:** Definition of Bayesian network nodes.

Variables	Node definition	Assignment
Gender	Gender	Female = 0, male = 1
Age (year)	Age	Age1 = 40 ~ 54 = 1, age2 = 55 ~ 64 = 2, age3 = ≥ 65 = 3
Exercise status	Exercise	Frequently = 1, occasionally = 2, little = 3
Smoke	Smoke	No = 0, yes = 1
Drink	Drink	No = 0, yes = 1
Hyperuricemia	Hyperuricemia	No = 0, yes = 1
High Hemoglobin A1C	High HbA1c	No = 0, yes = 1
Functional loss	Functional loss	No = 0, yes = 1
Body mass index level	BMI level	Normal = 1, thin = 2, overweight or obesity = 3
Progression status of metabolic syndrome	Progression status of MetS	Remain unchanged = 0, forward progression = 1
Recovery status of metabolic syndrome	Recovery status of MetS	Remain unchanged = 0, reverse recovery = 1

Bayesian network is a probabilistic graphical model, used to solve unqualified problems and visually describe complex relationships between multiple variables. It divided into two parts, a directed acyclic graph consisting of multiple nodes representing variables and directed acyclic edges reflecting the causal relationships of different nodes, where directed edges represent dependency or causality, and nodes connected by no edges are conditionally independent from each other, thus visually expressing the causal relationships between events; The other is the conditional probability table, which defines for each node the distribution of its variables in the network and enables a more accurate portrayal of the specific values of the dependencies between variables from a mathematical probability point of view.

The construction of the model usually includes structure learning and parameter learning^39^. Structure learning is used to determine the topology of Bayesian networks with directed acyclic graphs(DAG), usually be constructed through a priori knowledge and data learning, this study adopts a hybrid approach: combining a priori knowledge and data learning, it can be based on a priori knowledge, in the process of data learning, remove some redundant edges to simplify the structure, add directed edges to better fit the real problem, and take into account the efficiency and reasonableness of constructing the network structure. The number of Bayesian network structures that a data set with n variables in the DAG may contain is:

$$f\left(n\right)=\sum_{i=1}^{n}{(-1)}^{i+1}\frac{n!}{\left(n-1\right)!n!}{2}^{i(n-1)}f\left(n-1\right)$$

Bayesian network parameter learning means learning from the data to obtain the conditional probability distribution of each node based on determining the network structure. This study proposes to use the bnlearn package in R to obtain the network parameters by performing Bayesian parameter estimation using the Dirichlet distribution. $\theta =\left\{{\theta }_{ijk}|i=1,\dots ,n;j=\mathrm{1,2},\dots ,q;k=1,\dots ,r\right\}$, ${\theta }_{ij}$ denotes ${\theta }_{ij1},{\theta }_{ij2},\dots ,{\theta }_{ij{r}_{i}}$, ${\theta }_{i}$ denotes ${\theta }_{i1},{\theta }_{i2},\dots {\theta }_{i{q}_{i}}$, ${\theta }_{i}$ denotes the parameters of $P\left({x}_{i}|pa\left({x}_{i}\right)=j\right)$, $P\left({x}_{i}|pa\left({x}_{i}\right)\right)$ and $P\left({x}_{i}|pa\left({x}_{i}\right)=j\right)$ are, respectively, the conditional probability distribution of ${x}_{i}$ and all distributions of distributions, Estimates of the parameters can be calculated:

$${\theta }_{ijk}=\int P\left({\chi }_{i}=k|pa\left({\chi }_{i}\right)=j,{\theta }_{ijk}\right)P\left({\theta }_{ijk}|D\right)d{\theta }_{ijk}=\int {\theta }_{ijk}P\left({\theta }_{ijk}|D\right)d{\theta }_{ijk}$$

The sample size is important for the construction and robustness of the model, and in this study the sample size is much larger than the number of variables, as scholars say in this case the results obtained from the Bayesian network will be more stable^40^. In addition, to obtain a stable and valid DAG, using the bootstrap method to repeat the sampled data 10,000 times to reduce the influence of locally optimal (but globally suboptimal) individual DAGs on the results, the 10,000 bootstrap networks were averaged using the averaged network function, with each arc in the averaged DAG being present within a threshold of at least 85%, where the direction was given a threshold of 50%^41^.

### Bayesian network inference

Bayesian network inference can be achieved by calculating the probability of an event occurring based on the network structure and known evidence through the joint probability distribution formula. The magnitude of the change in probability can suggest the magnitude of the causal effect and can reveal the factor with a large change in effect, which is the key factor in the causal chain, in order to analyze and infer its causal mechanism. Based on Bayesian network learning, this study used GeNIe software to draw Bayesian networks and conditional probability distribution tables and perform causal and evidential inference based on them to analyze the size of the causal effect between the influencing factors and the progression and recovery of MetS.

Causal inference: the inference from cause to effect that infers the ending from the cause. Given a cause, a Bayesian formula is used to calculate the probability of the occurrence of the outcome. That is, instantiating the direct and indirect influences and observing changes in the rate of development or reversal of the MetS; Evidential reasoning: the cause-and-effect reasoning in which the cause is inferred from a known outcome. When the outcome is set as the MetS progression or reversal, the change in the probability of the influencing factor is calculated by Bayesian formula inference, and the cause of its generation is inferred, as well as the effect size.

### Statistical analysis

Data were managed with Excel 2016. R 4.0.2 software was used for statistical description and statistical analysis. The “bnlearn” package is used for Bayesian network learning, where the structure of the Bayesian network is learned using the hill-climbing algorithm combined with prior knowledge, and the parameters are learned using the Bayesian estimation method. GeNIe 2.3 software is used to visualize the Bayesian network and the conditional probability distribution table, and on this basis, Bayesian formulas are repeatedly used to perform causal and evidential inference on the network respectively.

### Ethics approval and informed consent

The CHARLS survey was approved by the Institutional Review Board of Peking University, China (IRB00001052-11014 and IRB00001052-11015).

## Data Availability

All data from the CHARLS is publicly available. This data can be found on http://charls.pku.edu.cn/en. The current study is a secondary analysis of public data of CHARLS.
